# Dietary moderately oxidized oil induces expression of fibroblast growth factor 21 in the liver of pigs

**DOI:** 10.1186/1476-511X-11-34

**Published:** 2012-03-06

**Authors:** Juliane Varady, Robert Ringseis, Klaus Eder

**Affiliations:** 1 Institute of Animal Nutrition and Nutrition Physiology, Justus-Liebig-University Giessen, Giessen, Germany

**Keywords:** Fibroblast growth factor 21, Liver, Peroxisome proliferator-activated receptor α, Pig, Oxidized fat

## Abstract

**Background:**

Fibroblast growth factor 21 (FGF21), whose expression is induced by peroxisome proliferator-activated receptor α (PPARα), has been recently identified as a novel metabolic regulator which plays a crucial role in glucose homeostasis, lipid metabolism, insulin sensitivity and obesity. Previous studies have shown that administration of oxidized fats leads to an activation of PPARα in the liver. Therefore, the present study investigated the hypothesis that feeding of oxidized fats causes an induction of FGF21 in the liver.

**Methods:**

Twenty four crossbred pigs were allocated to two groups of 12 pigs each and fed nutritionally adequate diets with either fresh rapeseed oil or oxidized rapeseed oil prepared by heating at a temperature of 175°C for 72 h.

**Results:**

In pigs fed the oxidized fat mRNA abundance and protein concentrations of FGF21 in liver were significantly increased (*P *< 0.05), and the protein concentrations of FGF21 in plasma tended to be increased (*P *< 0.1) in comparison to control pigs. Moreover, pigs fed the oxidized fat had increased transcript levels of the PPARα target genes acyl-CoA oxidase, carnitine palmitoyltransferase-1 and novel organic cation transporter 2 in the liver (*P *< 0.05), indicative of PPARα activation.

**Conclusion:**

The present study shows for the first time that administration of an oxidized fat induces the expression of FGF21 in the liver, probably mediated by activation of PPARα. Induction of FGF21 could be involved in several effects observed in animals administered an oxidized fat.

## Background

Fibroblast growth factor 21 (FGF21) has been recently identified as a novel metabolic regulator which plays a crucial role in glucose homeostasis, lipid metabolism, insulin sensitivity and obesity [1-3]. The physiologic importance of FGF21 is evident from studies showing that systemic administration of FGF21 reduces serum and liver triacylglycerol (TAG) concentrations, body weight and obesity in obese mice [4-6]. Hepatic expression of FGF21 is strongly up-regulated during fasting and is rapidly suppressed by refeeding, indicating a role of FGF21 in fasting-induced response [1-3]. In the liver, FGF21 stimulates hepatic lipid oxidation, ketogenesis and gluconeogenesis [1]. Recently, it has been found that expression of FGF21 is induced by peroxisome proliferator-activated receptor α (PPARα), a transcription factor which is activated during fasting by non-esterified fatty acids released from adipose tissue or by administration of synthetic or natural PPARα ligands such as fibrates or various types of fatty acids [1,3,7,8].

Oxidized lipids as components of heated or fried foods play an important role in nutrition in industrialized countries [9]. The ingestion of oxidized fats is generally regarded as detrimental for health. However, some studies in rodents have shown that oxidized fats could also exert some beneficial effects on metabolism such as a reduction of plasma and liver TAG and cholesterol concentrations, a reduction of obesity and even an inhibition of atherosclerosis [10-16]. Moreover, it has been demonstrated that dietary oxidized fats can activate PPARα in the liver [13,17-20].

Based on the finding that FGF21 is a target gene of PPARα, we tested the hypothesis that feeding an oxidized fat causes an induction of the expression of FGF21 in the liver which in turn raises the possibility that this hormonal factor is involved in several of the effects exerted by oxidized fats. For this end, we used samples from a recently performed experiment with pigs which received a moderately oxidized vegetable oil [21].

## Materials and methods

### Animals and diets

The samples used in this study were taken from animals of an experiment which has been recently reported in detail [21]. The experiment included 24 six week old pigs which were individually kept under adequate climate conditions. The pigs were allotted to two groups of 12 each. They were fed a nutritionally adequate diet which was composed of the following ingredients (in g/kg diet): Wheat (181.9), barley (100), soybean meal (containing 44% crude protein, 350), wheat bran (146), oil (150), choline chloride (containing 50% choline, 1.5), calcium hydrogen phosphate dehydrate [9], mineral and vitamin premix (40), L-lysine (containing 50.7 lysine, 16.5), DL-methionine (3.55), L-threonine (3.36), L-tryptophane (0.5). Concentrations of crude nutrients and vitamins and minerals were in accordance with recommendations for growing pigs [22]. The diets contained 16.4 MJ metabolizable energy, 224 g crude protein and 150 g of either a fresh or an oxidized oil per kg. As a source of oxidized oil, rapeseed oil which was heated in a domestic fryer at a temperature of 175°C for 72 h was used. As a source of fresh oil, a mixture of fresh rapeseed oil and fresh palm oil (91.6:8.4, w/w) was used. This fat mixture was used in order to equalize the fatty acid composition of the two oils (since the heating process caused a partial loss of PUFA in the rapeseed oil). Because the frying process caused a loss of tocopherols in the rapeseed oil, the vitamin E concentration of the oxidized fat was adjusted to that of the fresh fat by supplementation with all-rac-α-tocopheryl acetate (the biopotency of all-rac-α-tocopheryl acetate is considered to be 67% of that of α-tocopherol). The vitamin E concentration in the fresh fat diet was 98 mg α-tocopherol per kg diet, and that of the oxidized fat diet was adjusted accordingly. Concentrations of the major fatty acids and of some lipid peroxidation products in the fresh fat mixture and the oxidized fat are shown in Table 1.

**Table 1:** Fatty acid composition and concentrations of lipid peroxidation products in the experimental fats

	Fresh fat	Oxidized fat
**Fatty acid composition (g fatty acid/100 g total fatty acids)**		
16:0	9.3	6.8
18:0	2.5	2.2
18:1	55.4	60.5
18:2 (n-6)	21.9	21.1
18:3 (n-3)	7.0	5.4
**Lipid peroxidation products**		
Polar compounds (%)	2.82	23.1
TBARS (mmol/kg)	3.24	3.39
Peroxides (mEq O_2_/kg)	0.63	7.39

In order to avoid potential differences in food intake, due to adverse sensoric properties of the oxidized fat, a controlled feeding system was applied in which pigs were offered the same amount of diet during the 29 days feeding period. Water was available *ad libitum *from nipple drinkers during the entire experiment. All experimental procedures described followed established guidelines for the care and handling of laboratory animals and were approved by the local Animal Care and Use Committee (Regierungspräsidium Giessen; permission no: GI 19/3 No. 49/2010).

### Sample collection

After completion of the feeding period the animals were anaesthesised and exsanguinated 2.5 h after their last meal. Blood was collected into heparinized polyethylene tubes and plasma was subsequently obtained by centrifugation of the blood (1100 x *g*, 10 min, 4°C). Tissue samples from liver were dissected and stored at −80°C until analysis.

### Quantitative RT-PCR (qPCR)

The mRNA expression levels in the liver were determined as described recently in detail [21,23]. Features of primer pairs of reference and target genes, reference gene-stability measure *M *and data on qPCR performance for each gene measured in the liver are listed in Table 2.

**Table 2:** Characteristics and performance data of the primers used for qPCR analysis and reference gene-stability measure *M*

Gene	Forward primer (from 5' to 3') Reverse primer (from 5' to 3')	PCR product size (bp)	NCBI GenBank	Slope	R^2^	Efficiency	*M *value
*Reference genes*							
ATP5G1	CAGTCACCTTGAGCCGGGCGA TAGCGCCCCGGTGGTTTGC	94	NM_001025218.1	−0.2661	0.9981	1.85	0.036
GPI	CACGAGCACCGCTCTGACCT CCACTCCGGACACGCTTGCA	365	NM_214330.1	−0.2557	0.9964	1.80	0.034
RPS9	GTCGCAAGACTTATGTGACC AGCTTAAAGACCTGGGTCTG	327	CAA23101	−0.2705	0.9994	1.86	0.036
β-Actin	GACATCCGCAAGCACCTCTA ACATCTGCTGGAAGGTGGAC	205	NM_001167795	−0.2637	0.9979	1.84	0.046
SDHA	CTACGCCCCCGTCGCAAAGG AGTTTGCCCCCAGGCGGTTG	380	DQ402993	−0.2551	0.9986	1.80	0.041
*Target genes*							
FGF21	CGATACCTCTACACGGATGA CGTTGTAGCCATCCTCAAGT	158	NM_001163410.1	−0.2773	0.9968	1.88	-
HMGCS2	ATGGAACGCACGCAGCTCCC TCAGGCCCAACCAGCATGGC	323	NM_214380.1	−0.2528	0.9983	1.79	-
ACO	CTCGCAGACCCAGATGAAAT TCCAAGCCTCGAAGATGAGT	218	AF185048	−0.2495	0.9986	1.78	-
L-CPT1	GCATTTGTCCCATCTTTCGT GCACTGGTCCTTCTGGGATA	198	AF288789	−0.2654	0.9986	1.84	-
OCTN2	TGACCATATCAGTGGGCTA AGTAGGGAGACAGGATGCT	384	XM_003123912	−0.2517	0.998	1.79	-

### Determination of FGF21

Concentrations of FGF21 in plasma and liver were quantified by an ELISA assay kit highly specific to porcine FGF21 (No. E92918Po, Uscn Life Science Inc., Wuhan, China) according to the manufacturer's instructions. For the measurement of FGF21 in liver, approximately 0.4 g of liver was homogenized in 3.6 mL of PBS (71.5 mM NaCl, 99.9 mM Na_2_HPO4*2H_2_O, 26.8 mM KCl, 17.6 mM KH_2_PO_4_). Homogenates were centrifuged (4.000 x *g*, 20 min, 4°C) and the supernatants were used for determination of FGF21 after dilution (1 + 1, vol/vol) with PBS. Concentrations of FGF21 in liver homogenates were related to the protein concentrations in the homogenates as determined by the bicinchoninic acid (BCA) protein assay kit (Interchim, Montluçon, France) with BSA as standard.

### Determination of 3-hydroxybutyrate

Concentration of 3-hydroxybutyrate (3-HBA) in plasma was determined using an enzymatic reagent kit (Wako Chemicals GmbH, Neuss, Germany). In this assay, 3-HBA in the sample is oxidized in the presence of 3-hydroxybutyrate dehydrogenase and thio-NAD. 3-HBA is assayed by measuring the rate of thio-NADH production spectrophotometrically at a wavelength of 405 nm.

### Statistical analysis

Treatment effects were analyzed with one-way ANOVA using the Minitab Statistical software Rel. 13.0 (Minitab, State college, PA, USA). Statistical significance of differences of the mean values of the two groups of pigs was evaluated using Student's t test. Means were considered tendentially different at *P *< 0.1 and significantly different at *P *< 0.05.

## Results

### Concentrations of FGF21 in liver and plasma

**Figure 1:**
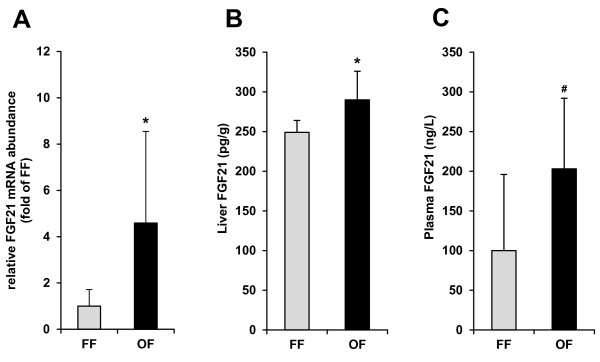
**Relative mRNA abundance of FGF21 in the liver (A) and protein concentration of FGF21 in the liver (B) and plasma (C) of pigs fed either a fresh fat or an oxidized fat**. Bars represent mean ± SD (n = 12/group), and are expressed as fold of fresh fat group. *Significantly (*P *< 0.05) and ^#^in tendency (*P *< 0.1) different from pigs fed the fresh fat. FF, fresh fat group; FGF21, fibroblast growth factor 21; OF, oxidized fat group

Pigs fed the oxidized fat had an increased relative mRNA abundance of FGF21 in the liver and an increased concentration of FGF21 in the liver compared to control pigs fed the fresh fat (*P *< 0.05, Figure 1). Plasma concentration of FGF21 in the pigs fed the oxidized fat was twice as much as in the pigs fed the fresh fat; however, the difference was not statistically significant due to a great standard deviation of the concentrations (*P *< 0.1, Figure 1).

### Expression of PPARα responsive genes in the liver

**Figure 2:**
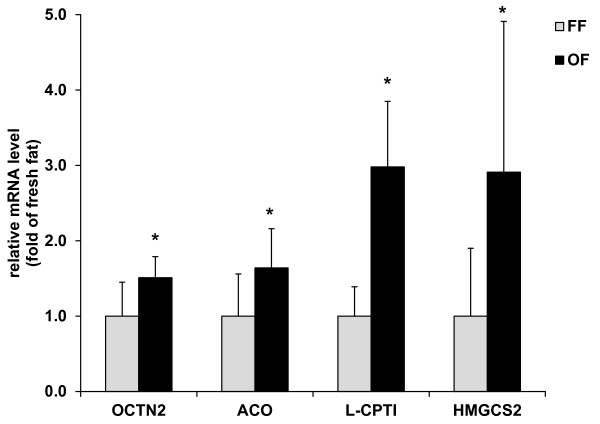
**Relative mRNA abundance of OCTN2, ACO, L-CPT1 and HMGCS2 in the liver of pigs fed either a fresh fat or an oxidized fat**. Bars represent mean ± SD (n = 12/group), and are expressed as fold of relative mRNA abundance of the fresh fat group. *Different from pigs fed the fresh fat, *P *< 0.05. ACO, acyl-CoA oxidase; HMGCS2, mitochondrial β-hydroxymethylglutaryl-CoA synthase 2; L-CPT1, liver-type carnitine palmitoyltransferase 1; FF, fresh fat group; OCTN2, novel organic cation transporter 2; OF, oxidized fat group

Pigs fed the oxidized fat had increased relative mRNA abundance of various PPARα responsive genes such as acyl-CoA oxidase (ACO), liver-type carnitine palmitoyltransferase 1 (L-CPT1) or novel organic cation transporter 2 (OCTN2) (*P *< 0.05, Figure 2), indicating an activation of hepatic PPARα in these animals.

### Expression of hepatic mitochondrial β-hydroxymethylglutaryl-CoA synthase 2 and plasma 3-HBA concentration

Pigs fed the oxidized fat had an increased relative mRNA abundance of mitochondrial β-hydroxymethylglutaryl-CoA synthase 2 (HMGCS2) in the liver (*P *< 0.05, Figure 2) and an increased concentration of 3-HBA in plasma compared to control pigs fed the fresh fat (fresh fat: 11.8 ± 1.93 μmol/L; oxidized fat: 17.3 ± 7.35 μmol/L; *P *< 0.05).

## Discussion

The aim of this study was to test the hypothesis that a dietary oxidized fat induces the expression of FGF21. For this end, we used samples of a recent experiment in which pigs were fed rapeseed oil heated in a domestic fryer under practical conditions [21]. Consideration of the concentrations of lipid peroxidation products (peroxides, TBARS, polar compounds) indicated that the oil used in that study was moderately oxidized and would be acceptable for frying of foods in human nutrition. The control fat and the oxidized fat were equalized for their fatty acid compositions and their vitamin E concentrations, and feed intake and body weight body gains of the pigs did not differ between the two groups [21]. Therefore, we can exclude the possibility that the effects observed in this study could have been confounded by a different intake of fatty acids or vitamin E, or by a reduced growth rate. It is well known that over-night starvation leads to an activation of PPARα which in turn induces the expression of FGF21 [2,3]. As we were interested in the effects of an oxidized fat on the expression of FGF21 without the distorting effect of fasting induced activation of PPARα, we collected the samples of the pigs in the fed state.

According to the hypothesis of this study, we observed for the first time that administration of an oxidized fat induced a strong induction of FGF21 in the liver, as indicated by a 5-fold increase of the mRNA abundance of that hormone in the liver. Although there is no direct evidence for this, the induction of FGF21 in the liver was probably mediated by activation of PPARα. In order to assess activation of PPARα, we determined the expression of three known PPARα target genes, namely ACO, L-CPT1 and OCTN2. The finding that these genes were up-regulated in the pigs fed the oxidized fat indicates an activation of PPARα in these pigs. Activation of PPARα in the liver induced by administration of oxidized fats has been established in rats and pigs [13,17,19,24], although the effect in pigs in this regard is less pronounced than in rodents due to the fact that pigs have generally a lower expression of PPARα and the response of many genes to PPARα activation in pigs is much lower than in rodents [25,26].

FGF21 has been recognized as a hormonal regulator that reduces plasma glucose and TAG concentrations, enhances insulin sensitivity, and inhibits development of obesity and hepatosteatosis [4-6]. Moreover, induction of FGF21 in the liver stimulates hepatic lipidnists, indicating that FGF21 and PPARα have several overlapping effects. Indeed, there are several other metabolic effects induced by both, FGF21 and PPARα agonists. For example, administration of both, PPARα agonists such as fibrates and FGF21 lowered LDL-cholesterol, raised HDL-cholesterol, improved insulin sensitivity and prevented diet-induced obesity in rhesus monkeys and rodents [4,27-30]. Due to this striking overlap in activities, it has been suggested that FGF21 contributes to many of the actions of PPARα agonists.

Studies in rodents have shown that many of the effects induced by administration of FGF21 such as a reduction of liver and plasma TAG concentrations [10-12,18,19,31], a reduction of plasma cholesterol [10,13,31-33], an increase in HDL-cholesterol concentration [31], a reduction of adipose mass [15], or an increase of insulin sensitivity [34] are also induced by dietary oxidized fat. Previous studies have shown that dietary oxidized fats can even prevent an alcohol induced steatosis or the development of atherosclerosis [16,35]. In this study, we did not measure parameters of lipid or glucose metabolism. However, recent studies have also shown that administration of a moderately oxidized fat lowers plasma and LDL cholesterol concentrations and plasma TAG concentration [36,37] and increases plasma 3-HBA concentration in pigs [24]. These studies indicate that administration of an oxidized fat exerts also effects on lipid metabolism in pigs. Although concentrations of FGF21 in plasma and liver were less increased than FGF21 mRNA abundance by administration of the oxidized fat, it is likely that at least a part of the metabolic effects observed in animals fed an oxidized fat might be induced by FGF21. Up-regulation of HMGCS2, the key enzyme of ketogenesis, and an increased plasma concentration of 3-HBA observed in the pigs fed the oxidized fat in the present study, might also be - at least in part - induced by FGF21 which is regarded as key regulator of ketogenesis [2]. Given the similarities between pigs and humans with respect to expression of PPARα [26] as an important regulator of FGF21 expression, the possibility exists that a dietary regime rich in fried foods containing oxidized fats induces hepatic expression of FGF21 in humans.

In conclusion, the present study shows for the first time that the ingestion of a moderately oxidized oil causes an induction of FGF21 in the liver of pigs, probably induced by activation of PPARα. This finding opens up the possibility that at least some of the effects observed in animals administered an oxidized fat are induced by FGF21.

## Abbreviations

ACO: Acyl-CoA oxidase; ATP5G1: ATP synthase, H + transporting: mitochondrial Fo complex, subunit C1; 3-HBA: 3-hydroxybutyrate; HMGCS2: Mitochondrial β-hydroxymethylglutaryl-CoA synthase 2; FF: Fresh fat group; FGF21: Fibroblast growth factor 21; GPI: Glucose-6-phosphate isomerase; L-CPT1: Liver isoform of carnitine palmitoyl transferase 1; OCTN2: Novel organic cation transporter 2; OF: Oxidized fat group; PPARα: Peroxisome proliferator-activated receptor α; RPS9: Ribosomal protein S9; SDHA: Succinate dehydrogenase complex, subunit A.

## Competing interests

The authors declare that they have no competing interests.

## Authors' contributions

JV carried out the experiments and participated in the interpretation of the data and drafted the manuscript. RR participated in the design of the study and supervised the experiment and analysis. KE conceived of the study and its design, coordinated work, participated in the interpretation of the results, and helped to draft the manuscript. All authors read and approved the final manuscript.
